# Increasing User Involvement in Health Care and Health Research Simultaneously: A Proto-Protocol for "Person-as-Researcher" and Online Decision Support Tools

**DOI:** 10.2196/resprot.3690

**Published:** 2014-11-25

**Authors:** Mette Kjer Kaltoft, Jesper Bo Nielsen, Glenn Salkeld, Jack Dowie

**Affiliations:** ^1^Research Unit of General PracticeInstitute of Public HealthUniversity of Southern DenmarkOdenseDenmark; ^2^School of Public HealthFaculty of MedicineUniversity of SydneySydneyAustralia; ^3^Faculty of Public Health and PolicyLondon School of Hygiene and Tropical MedicineLondonUnited Kingdom

**Keywords:** user involvement, decision support, patient empowerment, Internet

## Abstract

**Background:**

User involvement is appearing increasingly on policy agendas in many countries, with a variety of proposals for facilitating it. The belief is that it will produce better health for individuals and community, as well as demonstrate greater respect for the basic principles of autonomy and democracy.

**Objective:**

Our Web-based project aims to increase involvement in health care and health research and is presented in the form of an umbrella protocol for a set of project-specific protocols. We conceptualize the person as a researcher engaged in a continual, living, informal “n-of-1”-type study of the effects of different actions and interventions on their health, including those implying contact with health care services. We see their research as primarily carried out in order to make better decisions for themselves, but they can offer to contribute the results to the wider population. We see the efforts of the "person-as-researcher" as contributing to the total amount of research undertaken in the community, with research not being confined to that undertaken by professional researchers and institutions. This view is fundamentally compatible with both the emancipatory and conventional approaches to increased user involvement, though somewhat more aligned with the former.

**Methods:**

Our online decision support tools, delivered directly to the person in the community and openly accessible, are to be seen as research resources. They will take the form of interactive decision aids for a variety of specific health conditions, as well as a generic one that supports all health and health care decisions through its focus on key aspects of decision quality. We present a high-level protocol for the condition-specific studies that will implement our approach, organized within the Populations, Interventions, Comparators, Outcomes, Timings, and Settings (PICOTS) framework.

**Results:**

Our underlying hypothesis concerns the person-as-researcher who is equipped with a prescriptive, transparent, expected value-based opinion—an opinion that combines their criterion importance weights with the Best Estimates Available Now for how well each of the available options performs on each of those outcomes. The hypothesis is that this person-as-researcher is more likely to be able to position themselves as an active participant in a clinical encounter, if they wish, than someone who has engaged with a descriptive decision aid that attempts to work with their existing cognitive processes and stresses the importance of information. The precise way this is hypothesis tested will be setting-specific and condition-specific and will be spelled out in the individual project protocols.

**Conclusions:**

Decision resources that provide fast access to the results of slower thinking can provide the stimulus that many individuals need to take a more involved role in their own health. Our project, advanced simply as one approach to increased user involvement, is designed to make progress in the short term with minimal resources and to do so at the point of decision need, when motivation is highest. Some basic distinctions, such as those between science and non-science, research and practice, community and individual, and lay and professional become somewhat blurred and may need to be rethought in light of this approach.

## Introduction

User involvement is appearing increasingly on the policy and action agendas of health care providers and researchers in many countries. Both “user” and “involvement” are terms broad enough to encompass a wide variety of interpretations [1-3] and to evoke a variety of proposals for how involvement can be encouraged, facilitated, and increased, regardless of interpretation. The belief is that user involvement will produce better health consequences for individual and community and will demonstrate greater respect for the basic principles of autonomy and democracy.

In discussing obstacles to such increased user involvement, the need to tackle professional attitudes, institutional barriers, and silo borders must also be emphasized [4-7]. However, some of the most fundamental barriers and borders remain largely untouched and beyond questioning, except by some at the margins of the discourse.

In our project to increase the involvement of persons in health care and health research, we find four fundamental distinctions that are problematic: (1) science and non-science, (2) research and practice, (3) group and individual, and (4) professional and lay. The four pairs are linked insofar as scientific research occurs overwhelmingly at the public group level, while professional practice, either at the individual or community level, is non-scientific. We use non-scientific in the sense that the actual application of scientifically established evidence can never be validated by the standards of science, let alone the application of beliefs or judgments. The claim that practice is evidence-based or science-based confirms, rather than contradicts, this.

Against the background of the revolution in electronic communications and computer competencies (providing widespread online access) and informatics and information storage (generating large amounts of accessible big data), we see our project, outlined here in the form of an umbrella protocol, as an addition to the variety of technologies available to optimize user involvement. But it represents a challenge to the systemic dichotomies above.

All four of the above distinctions are implicit in the activities of INVOLVE in the United Kingdom, an excellent example of an attempt to increase user involvement in health and health care *research,* in contrast to parallel attempts to increase user (ie, patient) involvement in health care *practice.* INVOLVE is a national advisory group that supports greater public involvement in the National Health Service (NHS), public health, and social care research. It is funded by and is part of the National Institute of Health Research (NIHR), which is in turn funded by the Department of Health and is tasked with sharing knowledge and learning on public involvement in research.

INVOLVE defines the public as “patients and potential patients; people who use health and social services; informal carers; parents/guardians; disabled people; members of the public who are potential recipients of health promotion programmes, public health programs and social service interventions; and organizations that represent people who use services”. Public involvement in research is conceptualized as “doing research ‘with’ or ‘by’ the public, rather than ‘to’, ‘about’ or ‘for’ the public”. INVOLVE distinguishes between three main levels of public involvement: (1) consultation (where researchers seek the views of the public on key aspects of the research), (2) collaboration (an ongoing partnership between researchers and the public throughout the research process), and (3) “publicly led” (where the public designs and undertakes the research and where researchers are invited to participate only at the invitation of the public) [8].

The split between scientist/researcher, practitioner/professional, and lay/public is clear throughout INVOLVE’s descriptions but nowhere more clearly than in the final point. We see it as significant that INVOLVE has chosen to use the collective term “the public”, rather than the individual term “the person”, even though the former is then defined almost exclusively in terms of the latter.

Among the other instantiations of user involvement, “user *controlled* research” is a clear example of a publicly led activity, but it has political ambitions well beyond that envisaged by INVOLVE [9] (see Textbox 1).

**User controlled research quoted from INVOLVE [9].**

-The main aim of such research is seen as liberatory; supporting the empowerment of research participants and the achievement of change in line with service users’ rights and self-defined needs and interests. Such user controlled research has generally been based on:

-social rather than medicalized individual approaches and understandings;

-the rejection of positivist claims to “objectivity”;

-and a commitment to personal, social and political change.

The concept of control in research is not a simple one. It may be defined in different ways and open to different interpretations. Service users and their movements, however, have identified user control as the defining characteristic of research which advances user knowledge, rights, and interests.

Community-based participatory research is less radical and more in accord with the collaborative category of INVOLVE in that it promotes a specific two-way flow of information within the research group: researchers provide information and tools to enable community members to carry out research and take action, and community members share their expert knowledge and local meanings with researchers to achieve mutual knowledge and solutions to practical problems [10,11].

Within the status quo, three types of reasons are typically given for involving users in research [12]:

Public involvement in health research is underpinned by epistemological, moralistic and consequentialist arguments. The epistemological argument states that health research can benefit from the experiential knowledge and personal insights of patients, carers and service users. The moralistic argument states that the public have a right to be involved in any publicly funded research that may impact on their health status or the services that they receive. Finally, the consequentialist argument states that public involvement helps to improve the quality, relevance and impact of health research.

We suggest that a second consequentialist argument is missing from this list, particularly relevant within the setting of person-centered care [13]. In the Web-based project introduced here, we conceptualize the person as a researcher who is engaged in a continual, living, informal “n-of-1”-type study [14] of the effects of different actions and interventions on their own health, including those that imply contact with health care services. We see their research as primarily carried out in order to make better decisions for themselves, but they may offer to contribute the results to the wider population, either because it could eventually lead to better, or better-evaluated, interventions for themselves or because it could contribute to some wider public health goal or the good of others.

Within the conceptualization of person-as-researcher, those who lack the capability to function as effective researchers should be supported in their efforts to achieve that capability [15] through measures to increase health decision literacy and numeracy, especially in disadvantaged populations [16]. While we agree wholeheartedly with this principle, we note that questions of how far this support should go and at what resource cost must be part of the overall discussion of allocating scarce resources within a community, including those given to formal research. Without this reality check, all recommendations within the “capabilities” discourse remain ethically impressive but practically empty. Our project is designed to make some progress in this direction possible in the short term with minimal resources and to do so at the point of decision need, when motivation is highest.

## Methods

### Overview

Our online decision support tools, delivered directly to the person in the community and openly accessible, are to be regarded as “research resources”. The tools take the form of interactive decision aids for a variety of specific health conditions, as well as a generic one that aims to support all health and health care decisions through its focus on key aspects of decision quality.

The tools focus directly on the person-as-researcher’s fundamental question, “What should I do?” This requires answers to the two subordinate questions: “What should I believe?” and “What do I prefer?” They generate an *opinion* that integrates a set of beliefs, in the form of the Best Estimates Available Now (BEANs) for the performance of the relevant options on criteria that matter to the person, with their preferences, expressed as relative importance weights for those criteria. The integration, by a simple and transparent expected value calculation, produces a set of scores for each option that constitute the opinion produced by the process—nothing more and nothing less.

For some criteria, the person is themselves the expert source of the BEANs, since they measure the impact of options on their personal life. The difficulty, burden, or bother associated with administration routes for medications or journeys to provider facilities are good illustrations of where different individuals may make very different BEAN assessments. All persons-as-researchers contribute their individual preferences to the opinion as criterion importance weights.

Many who consult the tools in the course of their research will be satisfied that they have received a personalized opinion for their own private use. But they can offer to contribute the results of their n-of-1 research to an n-of-n database, by registering with the site by named email and declaring any conflict of interest. Their name will appear in any publication based on the aggregation of the individual results, though personal results will never be displayed. They receive feedback as part of the research team.

It is vital to be absolutely clear on one fundamental principle: whether or not the person is assigned, or accords themselves, the status of patient in some other setting, they are involved in our project as a researcher and only as a researcher. And we repeat that this approach is proposed as *one* method to be included in the portfolio of interventions needed to meet the very broad target of increased user involvement in a heterogeneous community.

From this point on the paper takes the form of an umbrella protocol for the condition-specific studies that will implement our approach. It is therefore organized using the Populations, Interventions, Comparators, Outcomes, Timings, and Settings (PICOTS) framework [17].

### Populations

Our population consists of individuals researching their personal health using a more or less formal n-of-1 methodology to help decide among different health-related interventions and actions. They regard themselves as interacting with health care professionals and institutions as an individual researcher, even though they are customarily assigned the status of patient. Individuals who wish to see themselves purely as patients are advised that they may find our resources, designed to support the individual’s research for better decision making, inappropriate or unhelpful. But we hope they will proceed, subject to confirming acknowledgment of being seen in a researcher role. Those who wish to see themselves mainly, or exclusively, as patients will be well catered for by patient-centered shared decision making [18].

The focus is solely on research for better decision making about the individual’s care. There is increasing interest in user involvement in relation to community-level activities, such as the development, prioritization, and delivery of health care services; the evaluation of specific interventions in Health Technology Assessments; and the determination of reimbursability for drugs and devices [1]. These are outside the scope of our project, though the approach we suggest is modifiable to this type of policy decision.

Members of the community are entitled to adopt whatever position they wish in relation to their individual interactions with health care professionals and institutions. That includes their interactions involving decision making, subject to any legal requirements, including giving informed consent. Our decision resources are, however, designed explicitly for those who wish to be able to involve themselves in clinical decision making as persons who are empowered (emancipated, enabled, armed) by their prior research. They are also intended for those who wish to keep open such positioning as an option, even if it may not eventually be exercised.

Researching one of our relevant tools will yield an opinion, based on principles that they have accepted (for their research purposes) and inputs they have supplied. We assume that the person opts into obtaining the opinion as part of the research basis for their decision involvement and emphasize that they are free to reject its content or use it in any way they wish in any subsequent decision communication with a clinician. “Clinician” should be interpreted throughout to include nurses, other health professionals, and clinical teams. “Person” should be interpreted to include the person-defined significant others and any legal guardian or proxy.

### Interventions

#### Condition Decision-Specific Aids

Our condition decision-specific aids (eg, Should I have a prostate-specific antigen [PSA] screening test for prostate cancer? What treatment is there for my osteoarthritis?) have several characteristics that distinguish them from most other decision support tools [19,20]. We believe it is these features that carry the potential to increase user involvement, especially for the population defined above and in relation to the specified type of involvement.

While the increased scientific research on values and preferences needed for health decisions [21] proceeds, along with that on information and knowledge, clinical decisions are being made second by second. It would be wrong to say that much of the formal research being undertaken is “fiddling”, though increasing concern with waste in research suggests some of it is, and even that many of the results will eventually be proven wrong [22-24]. Metaphorically, Rome is smoldering while academics are learning, and we agree with Wears that “Nothing can be gained by further perseveration in asking why clinicians fail to adopt research recommendations. Progress may come from asking, instead, why research is failing to provide useful answers to questions important to clinicians” [25]. More importantly, we should be asking questions that are important to persons-as-researchers.

As a result, and as part of our work to improve decision quality in person-centered care, we publicly offer, as research resources, decision support tools that do not require answers to many of the fundamental questions being pursued in scientific research. This is in contrast to most of the decision aids and guidelines produced within both the evidence-based and shared decision-making philosophies, which emphasize current uncertainties, ignorance, and the need for caution. We believe vague urgings to “be cautious” are unproductive, unless accompanied by some operational guidance on *how* to be cautious, given a decision is to be made *now*. We therefore make our offers on the basis that the underlying theory and principles of the aids, as well as the nature and provenance of their empirical inputs, are made clear before any engagement with them (or buy-in) is possible. The user is required to have read and accepted the contents before proceeding. We therefore assume that they are making an informed *meta*-decision about whether to engage with the aid before any further involvement, even as a researcher. An involvement strategy that proceeds without this sort of high-level consent goes beyond “persuasion-as-simply-making-available” into covert nudging at best and coercive manipulation of choice at worst. It is ethically questionable [26-28].

The aids produce an opinion based on a prescriptive model for decision making in the form of Multi-Criteria Decision Analysis (MCDA). The opinion is “dually personalized” as it consists of the scores produced by combining (in an expected value calculation) the person’s percentage importance weights for the criteria important to them with the BEANs for the personalized performance of each option on each criterion. The aids make absolutely no claim to be descriptively based in human decision behavior [29]. In fact, in key respects, especially their numerical format and expected value basis, their descriptive inadequacies are a necessary condition of their having something new and important to offer [30]. The aids are presented with as much transparency as possible, in order for the person to be clear about the principles underlying the opinion that emerges. We emphasize that they can reject the opinion of the aid as a contribution to their research, having generated it, but advise that they should consider not even engaging with it if they disagree with the bases spelled out upfront.

While we refer to “preferences”, our precise term is “importance weights”. As with most other terms in this area, debate surrounds its meaning. We define importance weights simply as the normalized responses of a respondent asked “How important is [each criterion] to you on an 11-point scale ranging from 0=‘of no importance’ to 10=‘of extreme importance’?”. After the responses are transformed into weights adding to 100% by normalization, the respondent has the opportunity to use the cursor or touch to modify the bar-length representations presented on the screen. We regard this elicitation procedure as the only one that is practical, in comparison to the more complex, normatively appealing procedures such as standard gambles, time trade-offs, and swing weights, which we have tried and found operationally lacking [19]. We do not take any position on whether these importance weights meet anybody’s normative requirements for constituting “utilities”. The key point is again to make clear to the respondent that it is their importance weights, so defined, that are entered into the personalized opinion that the aid will produce for them as part of their research.

In regard to the performance rates for options on criteria, our tools are *not* designed primarily as information aids. They are therefore clearly different from most other aids that assume a better decision must be an informed decision. We do provide links to high-quality sources of information so that the person-as-researcher can opt in to them if they choose. But it is made clear that our primary aim is to provide information in the form of the BEANs for the performance of each option on each criterion. These are updated within a “living” philosophy [31] and reject any generic value-judgment based threshold (eg, *P*<.05) for what is usable in clinical decision making. In the absence of robust evidence, they may be best elicited by expert-based elicitation. The BEANs entered into the individual’s aid are personalized as much as possible on the basis of self-reported characteristics. Opt-in pop-ups provide the provenance of the BEANs, or links to their sources, and the person-as-researcher is free to follow these as further clues to trustworthiness. Why do we not regard these as vital to consult in order to benefit from the aids? Because we are aiming solely to provide an opinion based on an expected value calculation that synthesizes the BEANs with the person’s importance weights. Given this purpose, there is no need to communicate about the size or quality of the detailed BEANs in the way typically envisaged by those who see “risk communication” as a central task in informed decision support. Achieving success in this task is difficult [32], perhaps not surprising in the light of the failures of the educational and socialization systems to produce a health literate and numerate population. The only information our person-as-researcher needs to acquire is what the aid will provide and its bases—and what it does *not* offer.

However, there is an important exception. The person-as-researcher does have an important role in supplying, at the point of decision, the BEANs for criteria where they are the expert. This is notably the case regarding the impact of testing and/or treatment on the individual as a person or party to a relationship. The rating of the burden or bother associated with, for example, different modes of treatment delivery (eg, oral, topical, subcutaneous injection, intravenous infusion; home, clinic, hospital) will vary with an individual’s workloads and capacities [33]. Personalized elicitation of the BEANs for such criteria is therefore appropriate—not the use of group averages such as those produced by discrete choice experiments. Note that this rating role of the user is conceptually completely different from the role they play in assigning an importance weighting to such criteria, relative to all the others.

Uncertainty is dealt with by offering quality-weighted and unweighted opinions. We make clear that the quality adjustments in the former represent, no more and no less, the judgments of the quality of the BEANs made by the team responsible for their production.

**Figure 1:**
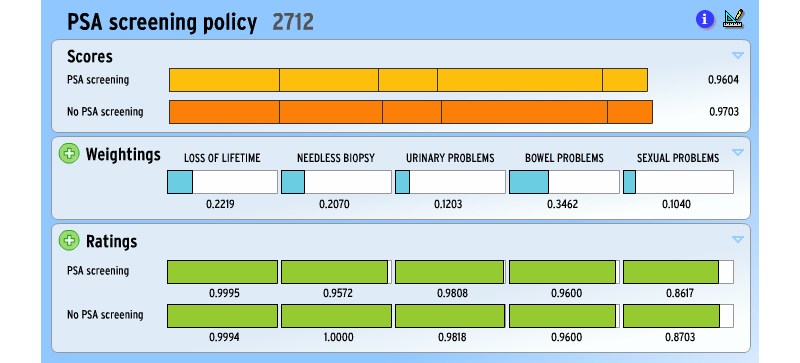
Example of PSA decision aid screen.

Our aids, such as “Should I have a PSA screening test for prostate cancer?” (Figure 1), are the product of teams of named health professionals, including clinicians. But we stress that the opinion emerging is not offered as, and should not be interpreted as, a medical opinion, legally speaking.

Most of the key requirements for accessibility, usability, and functionality of patient-centered decision support, whether they come in the form of computer-based decision aids or traditional professional interaction, apply equally to the design of aids to be presented as research resources [34-36]. Nevertheless, the re-conceptualization from patient to person-as-researcher does have major implications in the tone of address and register adopted. Most importantly, our decision aids should not be seen in any way as providing care, or as a way of delivering better care. Instead, they are intended simply as an optional resource available in the person’s own pursuit of the sources of better care. However, they also provide a way that users can add the results of their engagement to those of others, if they choose.

#### A Generic Decision Aid: MyDecisionQuality

User involvement is for a purpose, and our central aim is to improve decision quality. A measure of effectiveness in this regard is obviously needed.

MyDecisionQuality (MDQ) is a dually personalized decision quality instrument based (as are our condition decision-specific aids) on MCDA) [37]. The assessor (eg, the person) is responsible not only for (1) weighting the criteria of decision quality in terms of their relative importance, but also (2) rating the quality of a decision just made on the criteria. MDQ is generic in the sense that the criteria are phrased without reference to any particular decision or context. Information relating to the specific decision condition and setting must be provided (if at all) outside the MDQ instrument, such as in the wider condition-decision support resource where it will often be situated.

**Figure 2:**
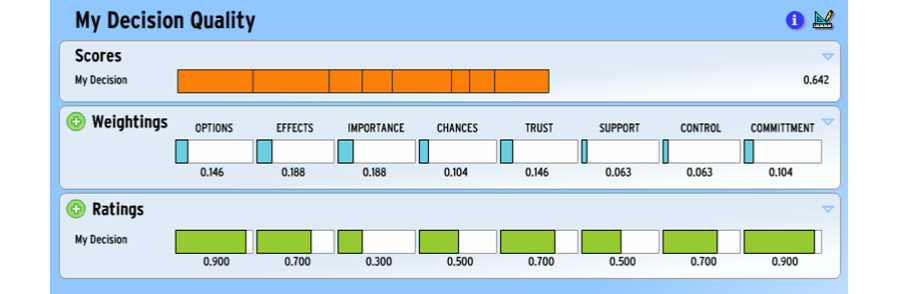
Example of MyDecisionQuality screen.

As with all implementations of the simple weighted-sum version of MCDA, MDQ combines a set of importance weights for multiple criteria with performance ratings for each option on these criteria and calculates the overall score as the expected value of these components. In the case of MDQ, the person’s weightings for the eight criteria of decision quality are elicited as early as possible in the decision-making process, and their ratings on how well the decision made performed on these criteria, as soon as possible after it was made. The MDQ score, unique to the person and to the particular occasion, is shown with the partial contributions of each criterion to it displayed in segments. Its weighting and rating are highlighted when the segment is touched or the cursor is rolled over it. An example is provided in Figure 2 and an illustrative video in Multimedia Appendix 1.

Apart from serving as an outcome measure for evaluating the decision-making process, MDQ represents an aid in itself and, being generic, can be used in conjunction with any of our condition decision-specific aids. Independent of any health care context or setting, MDQ alerts the person-as-researcher to one set of criteria for a good decision and asks them to express their importance weights for them. Even if these weights are not widely different from each other—not unusual since the criteria have been included because of their importance—the explicit attention given to them has the potential to influence the remainder of their decision-making research. Having rated the decision ex post on the same criteria, the person receives a dually personalized assessment of the quality of their decision. They are also provided with insight into the priorities for future quality improvement by being shown the quality gains possible from improved rating on each criterion, weightings unchanged. For example, in Figure 2 we can inform the person of the effect on their decision quality score of improving their rating on “Importance”, lowly rated at 0.3, given the relatively high weight of 0.188 they have assigned it. Achieving perfect rating on this criterion would increase their score by 0.7 x 0.188 or 0.132, equivalent to a 20% improvement. Feeding back the result of the same calculation for each of the criteria generates a personalized list of future priorities. Since the criterion “Effects” is already highly rated, it is unlikely to be high on this priority list, even though it has the same weight as Importance.

If an associated clinician completes the parallel MDQ instrument, the bases for a decomposable measure of concordance are established. A prescription for improved shared decision making in future is generated, if desired by both parties. It can help reduce the established differences in a person’s preferred and perceived participation in medical decision making [38].

MDQ can also serve as a patient-reported outcome measure (PROM), when the decision is conceptualized as one of the outcomes of a decision-making process. Or alternatively, it can be seen as a patient reported experience measure (PREM), which reflects their decision-making experience [39,40].

A bonus resulting from the use of both condition decision-specific and generic aids comes in the form of the enhanced and automatic documentation of the clinical decision-making process, given that the outputs can be saved by the person-as-researcher and incorporated into their provider’s and own health record/s, if desired.

### Comparators

Apart from a few aids also based on an implementation of MCDA (notably the Analytic Hierarchy Process), the vast majority of decision support tools on offer are not designed to produce an opinion in the form of numerical scores. They aim to support the person, normally regarded as a patient, by presenting information and value clarification exercises. They are then encouraged to make up their mind by taking into account and bearing in mind the pros and cons, without being offered explicit synthesizing principle or required to engage in numerical quantification or calculation. We can capture the difference from their aids succinctly by referring to the majority as being grounded in verbal multi-criteria decision deliberation as opposed to ours in numerical MCDA. Note that one of the key contrasts is expressed here as the verbal-numerical, rather than qualitative-quantitative one. All aids of both types are necessarily concerned with quantifying of magnitudes.

Our underlying hypothesis concerns the person-as-researcher who is equipped with a prescriptive, transparent, expected value-based opinion that combines their criterion importance weights with the BEANs for how well each of the available options performs on each of those outcomes. The hypothesis is that this person-as-researcher is more likely to be able to position themselves as an active participant in a clinical encounter, if they wish, than someone who has engaged with a descriptive decision aid that attempts to work with their existing cognitive processes and stresses the importance of information. Research that opens the “black box” of the clinical encounter [41,42] is revealing less and less impact from the latter approach to decision support. Most likely this is attributable to their failure to provide the person with powerful enough ammunition to move clinicians away from their preferred consultation structure and preferred course of action, reflecting tradition, training, and time constraints. This is particularly likely to happen in the situation where the evidence is low [43].

Apart from being provisional, the opinion from our aids will always be questionable by the normative standards built into many checklists for decision support tools [44,45]. We regard the relevant comparator as an empirical one, in the form of today’s clinician, and not abstract normative perfection. Experience so far shows there are many difficulties in carrying out genuinely unbiased empirical evaluations of person-centered decision aids in the clinical context—some methodological, some professional, and others legal.

### Outcomes

The black box metaphor is highly relevant in relation to the question that may be uppermost in some reader’s minds. What and where is the evidence of the impact of resources such as ours on any aspects of clinical decision making, notably user involvement and empowerment? A substantive, not merely rhetorical, response is to ask what and where the evidence is concerning the usual clinical decision-making process. Despite vast efforts to penetrate it, dating back to the pioneering work of Elstein [46], our aids will, by comparison, be shining white boxes. 

We note with interest that clinicians and health care institutions are largely free to introduce practice changes as “quality improvements” without citing any robust evidence base or reference to peer-reviewed evaluations. In person-centered care, it is surely appropriate to acknowledge individuals have the same right in regard to their health decisions and behaviors. Using online decision resources of our type, under their explicit ground rules, falls well within our concept of the person’s self-seeking quality improvement in health decision making, whether alone or in collaboration with clinicians.

Nevertheless, in the context of growing funding of research into interventions that (might) increase user involvement, serious evaluation is needed of both effectiveness and cost-effectiveness, with “multi-criteria” preceding “effectiveness” in both cases. Hence this high-level protocol, designed to set out the relevant issues. In our opinion, all user involvement interventions should be evaluated with a comparative methodology using the same empirical comparator, not a normative checklist. In other words, evaluation should be based on the same principles applied to drugs and devices. The relevant comparator will necessarily be a “usual practice” arm, and we welcome the opportunity to engage in an empirical comparison with all other proposed interventions on a “level playing field”. Unfortunately, experience shows the ethical and professional barriers to this may be considerable. Authorities contemplating evaluation and resourcing of alternative user involvement strategies should therefore be aware that the position they take on professional and ethical issues may well bias the result in a particular direction. That direction is more likely to be towards institutionalized forms of user representation and consultation than towards the more profound involvement envisioned within user controlled research, participant action research, and other emancipatory movements.

### Timing

Decision time is always *now*, so our tools are developed and maintained within a living philosophy [31], especially, in relation to the performance ratings, where living evidence-based network meta-analyses will need to be complemented by expert elicitation, to improve the quality of the BEANs for many person-important criteria. Elicitation could possibly be in the form of living expertise-based network meta-analyses [47].

### Settings

Our decision resources are designed to be practical for use at home in the community. This use may or may not be prior to some arranged or contemplated clinical consultation, depending on the individual person-as-researcher’s wishes. Their subsequent use in the clinical setting would be subject to the clinician’s agreement. Practicality in the home situation is the key to use of a resource designed to increase involvement. This will necessarily involve persons-as-researchers being allowed to make their own time and resource trade-offs in pursuing the complexity and depth offered.

## Results

As implied in the Comparator and Outcomes sections of the protocol, our underlying hypothesis concerns the person-as-researcher who is equipped with a prescriptive, transparent, expected value-based opinion—an opinion that combines their criterion importance weights with the Best Estimates Available Now for how well each of the available options performs on each of those outcomes. The hypothesis is that this person-as-researcher is more likely to be able to position themselves as an active participant in a clinical encounter, if they wish, than someone who has engaged with a descriptive decision aid that attempts to work with their existing cognitive processes and stresses the importance of information. The precise way this hypothesis is tested will need to be setting-specific and condition-specific, and these details will be spelled out in the individual project protocols.

## Discussion

### Other Considerations

The most advanced involvement of patient representatives in health research design and activity has been in OMERACT (Outcome Measures in Rheumatology) [48]. While important effects have been achieved, especially in adding person-important criteria such as fatigue to core outcome measures, the picture is not all rosy. Some participants in meetings have felt that “Dealing with hierarchical power relations and strongly opinionated professionals was experienced as mentally challenging. A recurring barrier reported by patients was a lack of feedback on provided contributions. At times they felt that their experiential knowledge was not accepted as a valid source for scientific research, nor seen as relevant compared to the expert knowledge of professionals” [48]. While this approach is likely to become more popular and effective, it will always be confined to a small number of patients. We seek much wider involvement through the open-access resources outlined in this paper.

Clinical decision making occurs as the final “bedside” stage of most translation models of the research-into-practice process. In many ways, it is the most complex stage to understand, to assess, and to intervene. We believe the Callard model is the most appropriate one for a person-centered health care system [49]. The user, now person-as-researcher, is separately placed in the middle of the model, rather than at the end of a translation pathway, or at one point in a cyclical translational system. Consequently they have direct impact on, and input into, all stages on the forward translation continuum from “bench to bedside”. In a small but significant modification to the Callard model, we suggest the person-as-researcher at the center is equipped with a decision support tool based on person-important criteria. The BEANs in their personalized resource represent the product of all necessary and practical forward translations needed at the point of decision, while the assessed quality of the BEAN for each cell constitutes the basis for backward translation to research priorities. In contrast (but not opposition) to the James Lind Alliance approach, which focuses on developing specific questions for researchers [50], priorities are indicated by the potential score gains for options from higher quality criterion ratings, given the criterion weights.

### Conclusions

Even a superficial overview of recent calls for increased user involvement in health care systems reveals a complex mix of motivations and interpretations. These are reflected in the diversity of terms and interpretations for both user (client, customer, patient, person) and involvement (participation, engagement, activation, emancipation). It is not surprising, then, that many and varied approaches to increasing user involvement have been canvassed, and implemented in some cases, without serious, comparative empirical evaluation.

In the light of this, our paper has had two purposes. The first explicit aim is to offer our specific person-as-(n-of-1) researcher approach that increases the individual’s involvement in health care practice and health care research simultaneously. The basis of the approach, through online interactive decision tools available as open access resources, differs significantly from most others on offer, and these differences extend to the theoretical and empirical bases of the aids. These have been described at length. The second implicit aim is to call attention to the need for careful and thorough specification, evaluation, and resourcing of programs or projects set up to achieve the broad aim of increased user involvement. Since there will be many considerations and stakeholders in play, both conceptual clarity and policy transparency make some form of multi-criteria analysis almost essential as policy decision support. A technique such as MCDA can ensure that the specifications of the options and criteria are precise and comprehensive. It will also ensure that the ratings of the options on each of the multiple criteria, which are likely to vary among stakeholders, are elicited and processed in a way that makes their provenance transparent.

Web-based decision resources such as those we produce can provide fast and efficient access to the results of slower thinking and encourage individuals to take a more involved role in their health production by viewing themselves as researchers involved in ongoing n-of-1 type studies.

Some basic distinctions, such as those between science and non-science, research and practice, community and individual, and lay and professional become somewhat blurred and will need to be rethought in the light of this approach. We encourage others to engage with us in this rethinking.

## Multimedia Appendix 1

Video demonstration of MyDecisionQuality.

## Abbreviations

BEAN: best estimate available now
MCDA: multi-criteria decision analysis
MDQ: MyDecisionQuality
OMERACT: Outcome Measures in Rheumatology
PICOT: Populations, Interventions, Comparators, Outcomes, Timings, Settings
PSA: prostate-specific antigen
